# Effects of Transcranial Direct Current Stimulation of the Posterior Parietal Cortex on Visual and Vestibular Function

**DOI:** 10.3390/neurosci7040080

**Published:** 2026-07-15

**Authors:** Sang Seok Yeo, Dong Hyun Byun, Fang He

**Affiliations:** 1 Department of Physical Therapy, College of Health Sciences, Dankook University, Cheonan-si 31116, Republic of Korea; yeopt@dankook.ac.kr; 2 Department of Special Creative Convergence, College of Rehabilitation Sciences, Daegu University, Gyeongsan-si 38453, Republic of Korea; bdh0529@hanmail.net; 3 Department of Public Health Science, Graduate School, Dankook University, Cheonan-si 31116, Republic of Korea

**Keywords:** posterior parietal cortex, balance, brain activity, brain stimulation

## Abstract

(1) Background: The effects of posterior parietal cortex (PPC)-targeted transcranial direct current stimulation (tDCS) on postural stability and cortical activity remain unclear. Therefore, this study aimed to investigate and compare changes in cortical activity and postural stability before and following tDCS conditions. (2) Methods: Eight right-handed adults completed a baseline assessment followed by three stimulation sessions: left-anodal/right-cathodal (L-A/R-C), left-cathodal/right-anodal (L-C/R-A), and sham on the PPC. The sessions were administered in a randomized Latin square design with a minimum 4-day washout period between each. At baseline and following each tDCS session, cortical activity was measured using functional near-infrared spectroscopy, and postural stability during a tandem stance was assessed utilizing the Balance Error Scoring System (BESS) and a force platform. (3) Results: Compared with the baseline measurements, significant deactivation in the right middle temporal gyrus (MTG) was observed following L-C/R-A stimulation. Furthermore, postural stability measures revealed significantly higher BESS error scores and greater sway length following L-C/R-A stimulation compared to both the baseline and L-A/R-C conditions. (4) Conclusions: Bilateral tDCS over the PPC differentially influences cortical activity and postural control depending on the stimulation polarity. Specifically, L-C/R-A stimulation was associated with impaired visual–vestibular integration and postural stability. These preliminary findings highlight the critical role of interhemispheric parietal balance in posture regulation and suggest that polarity-specific tDCS protocols may be important considerations for the future.

## 1. Introduction

Balance is maintained through the integration of multisensory inputs, including visual, vestibular, and somatosensory information. This integration process is orchestrated by the vestibular nuclei and parietal association areas of the cerebral cortex, which collectively generate adaptive motor responses [1,2]. Visual information is processed hierarchically from the primary visual cortex (V1) through the dorsal stream, encompassing the secondary visual cortex (V2), third visual area (V3), and middle temporal visual area (MT/V5), to the posterior parietal cortex (PPC) [3]. The processed sensory information then guides the motor system via descending neural pathways to generate appropriate postural responses for environmental adaptation [1].

Within this sensory integration process, the PPC serves as a critical neural hub, playing a vital role in postural control through its extensive connections with other cortical areas involved in multisensory processing [4]. The right PPC demonstrates increased cortical activity during visuospatial processing and attention allocation to environmental stimuli. It plays a crucial role in spatial navigation and the processing of optic flow signals during self-motion perception [5,6]. Clinical studies have indicated that patients with right parietal lobe lesions exhibit symptoms such as delayed functional recovery, dizziness, and seizures [7]. Conversely, the left PPC demonstrates selective activation in response to somatosensory feedback and vestibular inputs, contributing to body schema representation and sensorimotor integration during postural control [8,9]. Recent studies have found that deactivation of the left posterior parietal lobe inhibits the processing of somatosensory input, whereas patients with left parietal lobe lesions exhibit decreased visuomotor adaptation abilities [10,11].

Transcranial direct current stimulation (tDCS) is a non-invasive neuromodulatory technique capable of safely and selectively altering cortical excitability. It changes the local field polarity through the induction of transient electrical currents, facilitating the investigation of brain dysfunction pathogenesis and the development of neurorehabilitation protocols [12,13]. Regarding stimulation polarity, anodal tDCS increases resting membrane potential excitability, whereas cathodal stimulation decreases it [14].

Studies have reported that applying opposite-polarity stimulation over the parietal regions alters the interhemispheric parietal balance [12,15]. Furthermore, applying bilateral tDCS with opposite polarities over the parietal regions has been shown to modulate postural adaptation mechanisms, underscoring the critical role of the parietal cortex in processing multisensory information for postural control [16]. Recent tDCS studies have reported that cathodal stimulation of the left PPC leads to an asymmetric modulation of the vestibulo-ocular reflex and inhibits the parietal lobe’s processing of somatosensory information [10,17]. Moreover, the application of continuous theta burst stimulation (cTBS) to the right PPC has demonstrated significantly greater reductions in the center of pressure CoP sway variability compared to left PPC stimulation [18].

Despite these findings, studies examining the specific relationships among parietal lobe dysfunction, motor control, and postural stability remain limited, and the effects of PPC-targeted tDCS on balance and cortical activity have not been thoroughly investigated. Therefore, this study aimed to investigate and compare changes in balance and cortical activity before and after the application of different tDCS conditions. We hypothesized that different bilateral tDCS polarity conditions would differentially modulate balance performance and cortical activity. Specifically, compared with baseline, left-cathode/right-anode stimulation would produce greater cortical deactivation and balance impairment, whereas left-anode/right-cathode stimulation would show a different pattern of cortical activation and balance. Sham stimulation would produce minimal changes.

## 2. Materials and Methods

### 2.1. Participants

This study was designed as an exploratory study to provide preliminary evidence regarding the effects of different bilateral PPC tDCS polarity configurations on cortical activity and postural control in healthy adults. Eight healthy, right-handed adults (four males, four females; aged 20–30 years) were recruited. The inclusion criteria were as follows: (1) no prior experience with electrical stimulation; (2) no medical history of visual or cognitive impairments; (3) no history of neurological, orthopedic, or other systemic medical conditions; and (4) the ability to walk, maintain balance, and perform activities of daily living independently. All participants provided written informed consent, and the study protocol was approved by the Institutional Review Board of Dankook University (DKU 2024-03-024-003).

### 2.2. Transcranial Direct Current Stimulation (tDCS)

**Figure 1:**
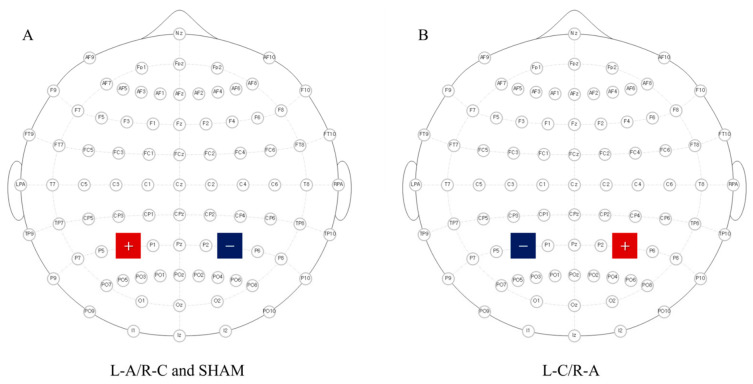
The tDCS montage and stimulation condition. The electrodes delivering tDCS were placed on the left and right posterior parietal cortex, over P3 and P4, respectively. The red square with a plus sign (+) represents the anode, and the blue square with a minus sign (−) represents the cathode. (**A**): left-anode/right cathode and sham condition stimulation, (**B**): left-cathode/right anode condition stimulation.

tDCS was administered using an ActivaDose II device (Caputron, New York, NY, USA) to modulate cortical excitability. The stimulation protocol consisted of a 2 mA constant current applied for 20 min, with 30 s ramp-up and 10 s ramp-down periods. Electrode placement followed the international 10-20 EEG system, utilizing two montage configurations targeting the bilateral PPC: (1) left-anodal/right-cathodal stimulation (L-A/R-C; anode: P3, cathode: P4) and (2) left-cathodal/right-anodal stimulation (L-C/R-A; cathode: P3, anode: P4). This montage has previously been shown to successfully induce parietal asymmetries [15]. For the sham condition, electrodes were positioned identically to the L-A/R-C condition (anode: P3, cathode: P4); however, the current was discontinued after 10 s to elicit initial sensory effects without providing sustained stimulation (Figure 1).

In a single-blind design, the participants were unaware of the stimulation conditions during each session, whereas the investigator was not blinded.

### 2.3. Measurement

#### 2.3.1. Functional Near-Infrared Spectroscopy (fNIRS)

**Figure 2:**
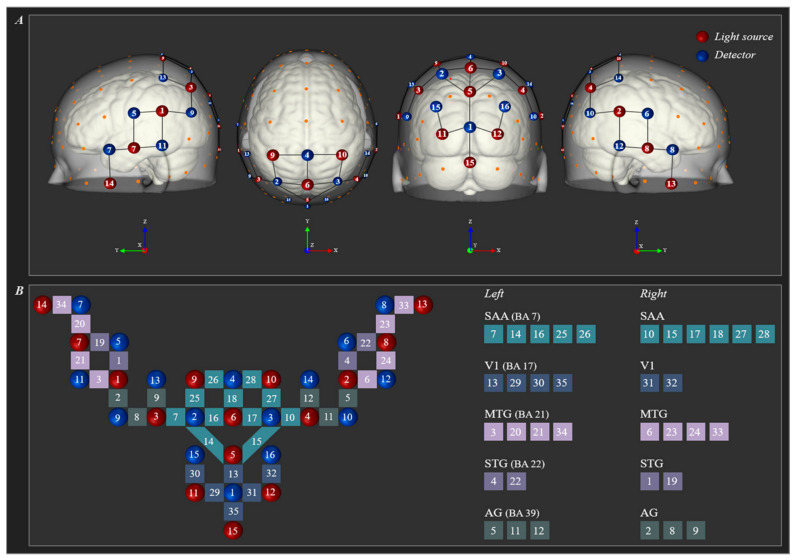
fNIRS optode placement and channel configuration. (**A**) fNIRS optodes placement: The fifteen red and sixteen blue circles represent the positions of the source and detectors, respectively. (**B**) Channel arrangement and region of interest; SAA: Somatosensory association area, V1: Primary visual cortex, MTG: Middle temporal gyrus, STG: Superior temporal gyrus, AG: Angular gyrus.

In this study, functional near-infrared spectroscopy (fNIRS) (NIRSport2, NIRx Medical Technologies LLC, Berlin, Germany) was used to evaluate changes in oxygenated hemoglobin (HbO) hemodynamic responses between the baseline and following stimulation for each tDCS condition [19]. Based on previous studies, the regions of interest (ROIs) were defined as the somatosensory association area (SAA) (Brodmann area (BA) 7), V1 (BA 17), middle temporal gyrus (MTG)(BA 21), superior temporal gyrus (STG)(BA 22), and angular gyrus (AG)(BA 39), as these areas are strongly associated with vestibular, visual, and multisensory integration [1,20]. The fNIRS system consisted of 15 sources and 16 detectors arranged with an inter-optode distance of 3 cm. Optical signals were recorded at two wavelengths (760 and 850 nm), yielding a total of 35 measurement channels (Figure 2).

#### 2.3.2. Balance Error Scoring System (BESS)

Postural stability was assessed using the Balance Error Scoring System (BESS), which evaluates postural control through the integration of somatosensory, visual, and vestibular inputs. The protocol comprised three distinct stance conditions: (1) double-leg stance with feet together and hands positioned on the iliac crests; (2) single-leg stance on the non-dominant lower extremity with hands in the same position; and (3) tandem stance with the non-dominant foot positioned posteriorly in a heel-to-toe alignment. Each stance configuration was performed under two surface conditions (firm and foam) with visual input eliminated (eyes closed). Performance was quantified by recording the number of errors committed during standardized 20 s trials. Error criteria were operationally defined as follows: opening the eyes, removing hands from the iliac crests, stepping, stumbling, or falling, elevating the heel or forefoot, abducting the hip beyond 30 degrees, or failing to return to the testing position within 5 s [21].

#### 2.3.3. Postural Control Task

Postural control parameters were analyzed using freeStep software (version 2.0; Sensor Medica, Rome, Italy) during tandem stance trials with eyes open. Postural control measurements were acquired via a calibrated force platform equipped with built-in pressure sensors. The measurement system operated at a sampling frequency of 100 Hz to capture the temporal and spatial characteristics of postural sway. Primary outcome variables included sway length (total path length of the CoP trajectory), ellipse surface (95% confidence ellipse area of CoP displacement), Delta X (maximum CoP displacement in the mediolateral direction), Delta Y (maximum CoP displacement in the anteroposterior direction), and average speed (mean velocity of CoP movement) [22]. All data acquisition and processing procedures were executed on a stable measurement platform to ensure the precision and reliability of the collected parameters.

### 2.4. Experimental Procedure

In the experimental protocol, participants completed a pre-tDCS measurement as a common baseline across all stimulation conditions, and subsequently underwent three stimulation sessions in a randomized Latin square design: L-A/R-C tDCS, L-C/R-A tDCS, and sham stimulation. The first session was conducted immediately following the baseline measurement, while each stimulation session was separated by a minimum 4-day washout period to prevent potential carryover effects and vestibular habituation. Baseline measurement and post-stimulation assessments included the measurement of cortical hemodynamic responses using fNIRS and the evaluation of postural control using the BESS and the freeStep platform. All participants were instructed to avoid excessive caffeine prior to the experiment. While individual fasting states and testing times varied naturally, all fNIRS assessments were immediately initiated following the tDCS sessions to minimize baseline physiological fluctuations.

During fNIRS measurements, a block design paradigm was adopted in the tandem stance condition with eyes open, including a low-demand baseline state (supported stance) and a high-demand task state (unsupported stance). Specifically, the session consisted of three 15 s task blocks of unsupported tandem standing, interspersed with and bracketed by four 15 s baseline blocks of supported tandem standing. Light physical assistance was provided by the examiner during the supported blocks to ensure postural stability.

### 2.5. Neuroimage Analysis

For task-related activation analysis, fNIRS data were processed using the NIRS-SPM toolbox (SPM8) within NIRSLab software (version 2019.04; NIRx Medical Technologies LLC, Berlin, Germany). Channels with poor signal quality were excluded based on a coefficient of variation (CV = standard deviation/mean)) threshold of 15%. The remaining signals were band-pass filtered (0.001–0.10 Hz) to reduce low-frequency drift and physiological noise, including cardiac and respiratory components, as well as Mayer wave fluctuations [23]. Subsequently, optical density data were converted into concentration changes of oxygenated hemoglobin (HbO) using the modified Beer–Lambert law. HbO was used for all subsequent analyses because it provides greater sensitivity and a higher signal-to-noise ratio than deoxygenated hemoglobin [24]. Task-related HbO responses were modeled using a general linear model (GLM) convolved with a canonical hemodynamic response function (HRF). A first-level (SPM-1, within-subject) analysis was performed to estimate channel-wise task-related activation using a supported stance (0)/ unsupported stance (1) contrast.

At the second level (SPM-2), within-subject analyses were conducted to assess and compare activation changes across baseline and post-stimulation measurements within each tDCS condition. Statistical parametric maps (t-maps) were generated based on these contrasts [25]. Given the exploratory nature of this study and the relatively small sample size, statistical significance was determined using an uncorrected *p*-value threshold of *p* < 0.05 at the channel level.

### 2.6. Statistical Analysis

Balance control parameters were analyzed using SPSS software (version 25.0; IBM SPSS, Chicago, IL, USA). The Shapiro–Wilk test was applied to assess the normality of the data distribution. The Friedman test was used to evaluate significant differences in balance parameters across the baseline measurement and the three tDCS conditions (L-A/R-C, L-C/R-A, and sham). When significant differences were detected, post hoc analyses were conducted using Wilcoxon signed-rank tests with Benjamini–Hochberg False Discovery Rate (BH-FDR) correction for multiple comparisons. Effect sizes were reported as Kendall’s W for Friedman tests and r (= |Z|/√N) for Wilcoxon signed-rank tests. Continuous variables were described as mean ± SD or median (interquartile range [IQR]). Statistical significance was set at *p* < 0.05.

## 3. Results

### 3.1. Demographic Data of Participants

The demographic characteristics of the participants are summarized in Table 1.

### 3.2. Cortical Activation Patterns During Postural Control Across Experimental Conditions

**Figure 3:**
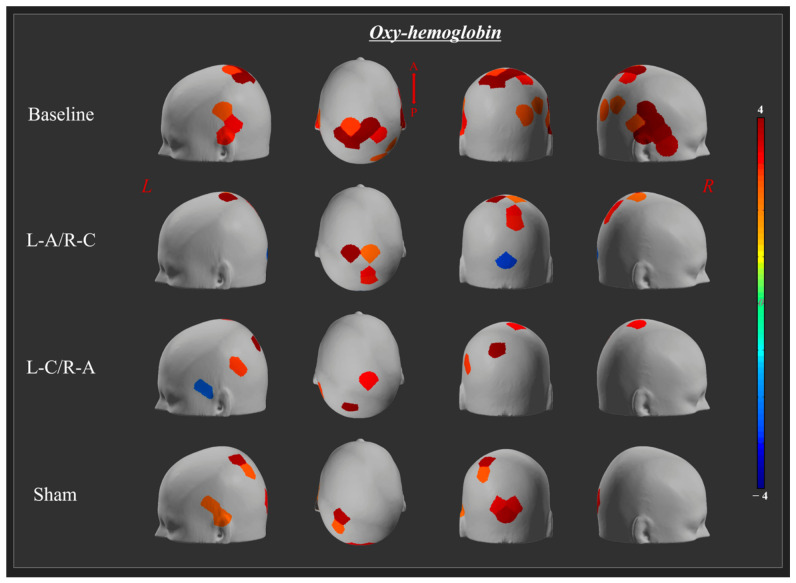
The t-maps of oxyhemoglobin (HbO) from the baseline measurement, following stimulation under L-A/R-C (left anode/right cathode), L-C/R-A (left cathode/right anode), and sham condition (GLM, uncorrected *p* < 0.05). L: left, R: right, A: anterior, P: posterior.

During the tandem stance, baseline measurements demonstrated significant activation in the bilateral SAA, STG, and MTG, as well as in the right V1 and AG (*p* < 0.05). Following L-A/R-C stimulation, significant activation was observed in the bilateral SAA, accompanied by significant deactivation in the left V1 (*p* < 0.05). Following L-C/R-A stimulation, significant activation was found in the bilateral SAA and left AG, whereas a reduced hemodynamic response was observed in the left MTG (*p* < 0.05). Under the sham condition, significant activation was observed in the bilateral V1, as well as in the left SAA, STG, and MTG (*p* < 0.05) (Table 2; Figure 3).

### 3.3. Differential Cortical Activation Between Experimental Conditions

**Figure 4:**
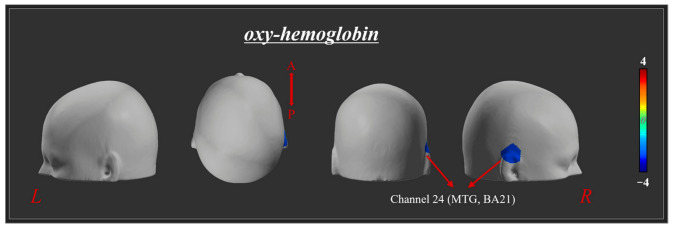
The t-statistic maps of oxy-hemoglobin (HbO) showing the contrast between the following stimulation under L-C/R-A condition and baseline measurement (uncorrected *p* < 0.05). L: left, R: right, A: anterior, P: posterior; MTG: middle temporal gyrus.

To further evaluate the effects of tDCS, pairwise comparisons were performed among the four experimental conditions. The t-values for all channels when comparing the L-C/R-A condition with the baseline measurement are detailed in Supplementary Table S1. Among these channels, significantly reduced activation in the right MTG (channel 24) was detected only following the L-C/R-A compared with the baseline measurement (*p* < 0.05) (Table 3; Figure 4). No other pairwise comparisons reached statistical significance (*p* > 0.05).

### 3.4. Comparison of Balance Parameters Between Sessions

A preliminary analysis confirmed that the chronological order of the stimulation sessions had no significant effect on the balance parameters (*p* > 0.05), indicating the absence of order or learning effects.

An analysis of the balance parameters revealed significant effects of tDCS on postural stability (Table 4). BESS scores and sway length exhibited significant differences across the stimulation conditions (*p* < 0.05), whereas the ellipse surface, average speed, Delta X, and Delta Y did not differ significantly (*p* > 0.05). Post hoc analyses revealed that the BESS scores were significantly higher in the L-C/R-A.

Post hoc analyses using BH-FDR correction revealed that BESS scores were significantly higher in the L-C/R-A condition compared to both the baseline (Z = −2.527, *p* = 0.012, r = 0.63) and the L-A/R-C (Z = -2.366, *p* = 0.018, r = 0.59) stimulation conditions. Similarly, sway length was significantly greater in the L-C/R-A condition compared to the baseline measurement (Z = -2.240, *p* = 0.025, r = 0.56). 

## 4. Discussion

In this study, we investigated the effects of bilateral tDCS over the PPC on cortical activity and postural control. Significant differences in cortical activity and balance parameters were observed across the various stimulation conditions. These findings provide preliminary evidence suggesting a potential causal role of parietal interhemispheric interactions in multisensory integration during balance control.

We observed distinct response patterns of cortical activation following different stimulation conditions during the tandem stance. In the baseline measurements, significant activation was observed in the bilateral SAA, STG, and MTG, as well as in the right V1 and AG, suggesting that these cortical regions are involved in postural control and multisensory integration during balance maintenance [22]. Compared to the baseline, L-C/R-A stimulation induced significant deactivation in the right MTG. This finding may reflect the opposing modulation of cortical excitability induced by tDCS, which could alter interhemispheric coordination and consequently influence the neural networks responsible for visual–vestibular integration [15,26,27]. In the absence of external support, postural control relies more heavily on visual and vestibular information, which requires precise interhemispheric coordination during tandem stance [28]. Altered interhemispheric coordination may impair the visuospatial processing and multisensory integration essential for maintaining postural control [29,30]. Therefore, the reduced activation observed in the right MTG may reflect less efficient visual–vestibular integration necessary for postural stability [10,31].

This reduced pattern may further reflect altered parieto-temporal network interactions, whereby the PPC normally exerts top-down modulatory control over visual information processing, thereby supporting effective visual–vestibular integration during balance control [32]. We hypothesize that the altered interhemispheric coordination may interfere with this modulatory control, contributing to the deactivation of the right MTG and compromising the visual information processing necessary for efficient visual–vestibular integration during postural control. Specifically, the right MTG plays a key role in self-motion perception and the maintenance of postural stability [10].

Interestingly, the observed cortical activation pattern did not fully conform to the conventional polarity-dependent effects often associated with tDCS. According to previous studies, tDCS over parietal areas can differentially modulate postural control depending on the availability of visual input, and PPC stimulation can alter visuospatial localization in complex, non-linear ways [17,33]. These results may be compatible with the principle of homeostatic plasticity, suggesting that excessive stimulation could induce compensatory neural responses aimed at maintaining network stability [34]. This interpretation highlights that, particularly in the context of higher-order cognitive functions, the relationship between stimulation polarity and behavioral outcomes may be more complex than what is predicted by simple excitation–inhibition models [27,31].

However, this interpretation remains speculative. While the right MTG deactivation following L-C/R-A stimulation may be consistent with homeostatic plasticity or compensatory neural activity aimed at maintaining network stability, we cannot exclude the influence of signal noise, potential inaccuracies in anatomical labeling, or stochastic resonance effects [35]. In addition, stochastic resonance has been proposed as an important mechanism underlying the balance-enhancing effects of noisy galvanic vestibular stimulation (nGVS). Although our study employed tDCS rather than nGVS, this mechanism provides an additional perspective for understanding neuromodulation-induced changes in multisensory processing and balance control [35,36].

An analysis of the balance parameters showed that BESS scores and sway length increased following L-C/R-A stimulation compared with both the baseline and L-A/R-C stimulation, indicating reduced postural stability during static balance. These findings may suggest that L-C/R-A stimulation, which was intended to inhibit the left PPC and facilitate the right PPC, may negatively affect postural control in healthy adults. The left PPC has been associated with the processing of somatosensory and vestibular inputs, while the right PPC shows increased activity during visuospatial processing and optic flow perception [8,37,38,39]. Cathodal stimulation of the left PPC may reduce the processing of somatosensory and vestibular inputs, thereby increasing reliance on visual information processed by the right PPC during balance control [39]. Previous studies have shown that left-hemisphere cathodal stimulation over P3 causes asymmetry in the vestibulo-ocular reflex, indicating that this stimulation reduces the PPC’s processing of vestibular information and the integration of sensory inputs [10,17].

Taken together, our findings suggest the differential roles of the left and right PPC in postural control. Because only L-C/R-A stimulation significantly impaired balance parameters, it suggests that the left PPC plays an important role in maintaining postural stability during a tandem stance. This observation is consistent with previous studies suggesting that the left PPC is specifically implicated in the processing of somatosensory feedback and the integration of vestibular inputs [18,40]. Interhemispheric parietal balance appears crucial for effective multisensory integration, and disrupting this balance through opposite-polarity stimulation may impair postural control, particularly when left PPC function is inhibited [12].

However, this study has several limitations that should be considered. First, the sample size was relatively small, and the age range of the participants was limited; tDCS effects may vary across different age groups. Second, fNIRS analyses relied on uncorrected statistical thresholds, meaning the possibility of false positives cannot be completely excluded. Additionally, the fNIRS system used in this study had two main technical constraints: it lacked short-separation channels, restricting our ability to isolate cortical signals from systemic noise (e.g., tDCS-induced scalp blood flow changes), and its limited penetration depth (1.5–2 cm) confined measurements to the superficial cortex. Third, conventional tDCS sponge electrodes lack spatial specificity, leading to diffuse current spread beyond the target PPC into adjacent cortical regions [41]. Fourth, this study utilized a single-blind design without a formal assessment of blind effectiveness. Although our reliance on purely objective physiological and biomechanical measures significantly mitigates the risk of experimenter bias, future large-scale trials should implement strict double-blind protocols to further ensure data robustness. Future studies with larger cohorts should employ high-definition tDCS, electric-field modeling, and multiple-comparison corrections to confirm and extend these exploratory findings.

## 5. Conclusions

In conclusion, our findings suggest that bilateral tDCS over the PPC influences cortical activity and postural control. Specifically, L-C/R-A stimulation was associated with decreased response in the right MTG and poor postural stability, emphasizing the critical role of interhemispheric balance in visual–vestibular integration and postural control. Furthermore, the findings suggest that postural control depends on balanced parietal function and that the relationship between stimulation polarity and behavioral outcomes is complex, potentially involving mechanisms of homeostatic plasticity. Although these findings should be interpreted with caution given the exploratory nature and sample size of the study, they provide preliminary evidence that stimulation polarity and site selection may be important considerations for future neuromodulation strategies and the development of personalized stimulation protocols.

## Figures and Tables

**Table 1:** Demographic characteristics of the groups.

Variable	Value
Age (years)	24.38 ± 2.00
Height (cm)	168.13 ± 8.54
Weight (kg)	62.63 ± 13.86
BMI (kg/m^2^)	21.96 ± 3.40
Sex	4 Female/4 Male
Dominant leg	8 Right

Mean ± SD.

**Table 2:** Significant channels and t-value for HbO responses in the baseline and after each tDCS condition stimulation.

Brain Region		Baseline	L-A/R-C	L-C/R-A	SHAM
Left	SAA (BA 7)	16 (4.08), 25 (3.91), 26 (2.51)	26 (4.15)	14 (3.97)	7 (2.37), 25 (3.65)
	V1 (BA 17)		35 (−2.49)		29 (3.14), 35 (3.58)
	MTG (BA 21)	3 (3.03), 21 (2.79)		20 (−2.22)	21 (2.24)
	STG (BA 22)	1 (2.28)			19 (2.23)
	AG (BA 39)			2 (2.58)	
Right	SAA (BA 7)	18 (4.02), 27 (2.94), 28 (6.17)	15 (2.80), 17 (3.07), 28 (2.24)	28 (3.07)	
	V1 (BA 17)	32 (2.34)			31 (3.29)
	MTG (BA 21)	6 (2.36), 23 (3.77), 24 (4.40), 33 (3.54)			
	STG (BA 22)	4 (4.31), 22 (6.33)			
	AG (BA 39)	11 (2.21)			

HbO: oxyhemoglobin, tDCS: transcranial direct current stimulation, L-A/R-C: left-anode/right cathode, L-C/R-A: left-cathode/right anode, SAA: somatosensory association area, V1: primary visual cortex, MTG: middle temporal gyrus, STG: superior temporal gyrus, AG: angular gyrus.

**Table 3:** Significant HbO response of L-C/ R-A condition stimulation compared with baseline measurement.

Stimulation	Brain Region	Channel	t
L-C/R-A	Rt middle temporal gyrus (BA 21)	24	−2.44

HbO: oxyhemoglobin, L-A/R-C: left-anode/right cathode, L-C/R-A: left-cathode/right anode, Rt: right.

**Table 4:** Changes in the balance parameter in the baseline measurement and after each condition of stimulation.

Balance Parameter	Baseline ^a^	L-A/R-C ^b^	L-C/R-A ^c^	SHAM ^d^	χ^2^	W	*p*	Post Hoc (r)
BESS	15.00 [13.25, 19.75]	16.00 [14.25, 20.25]	22.50 [16.50, 30.50]	13.50 [11.25, 21.00]	15.935	0.664	0.001 *	c>a (0.63) c>b (0.59)
Sway length (mm)	348.68 [336.58, 365.82]	423.67 [359.73, 521.16]	449.17 [349.71, 520.23]	372.93 [350.19, 460.36]	9.150	0.381	0.027 *	c>a (0.56)
Ellipse surface (mm^2^)	337.30 [283.31, 394.86]	467.47 [258.09, 803.69]	386.90 [223.14, 666.32]	341.88 [211.58, 512.39]	1.949	0.081	0.583	
Average speed (mm/s)	23.28 [21.76, 24.42]	28.37 [24.04, 34.84]	25.25 [22.38, 34.27]	24.92 [23.40, 30.83]	7.800	0.325	0.050	
Delta X (mm)	16.16 [12.74, 20.03]	16.64 [14.27, 21.33]	14.40 [10.32, 23.15]	16.59 [10.43, 20.52]	3.453	0.144	0.327	
Delta Y (mm)	35.25 [31.42, 40.57]	44.92 [30.53, 47.53]	41.40 [29.22, 60.42]	39.71 [24.78, 50.63]	2.85	0.119	0.415	

Median [interquartile range (IQR)]. *χ*^2^ and W represent the test statistic and Kendall’s W effect size for the Friedman test, respectively. r represents the effect size for the Wilcoxon signed-rank post hoc comparisons. ^a^: baseline; ^b^: L-A/R-C (left-anode/right cathode); ^c^: L-C/R-A (left-cathode/right anode); ^d^: SHAM. BESS: Balance Error Scoring System. * *p* < 0.05.

## Data Availability

The data presented in this study are available from the corresponding author upon reasonable request. The data are not publicly available due to privacy and ethical restrictions involving human participants.

## Supplementary Materials

The following supporting information can be downloaded at: https://www.mdpi.com/article/10.3390/neurosci7040080/s1, Table S1: HbO response of L-C/ R-A condition stimulation compared with baseline measurement.

## Abbreviations

The following abbreviations are used in this manuscript:

tDCS	Transcranial Direct Current Stimulation
PPC	Posterior Parietal Cortex
fNIRS	Functional Near-Infrared Spectroscopy
HbO	Oxygenated Hemoglobin
