# Supplementary material for: Effects of Transcranial Direct Current Stimulation of the Posterior Parietal Cortex on Visual and Vestibular Function
Source: NeuroSci. 2026 Jul 15;7(4):80. doi: 10.3390/neurosci7040080 (PMC13398033; doi:10.3390/neurosci7040080)
Supplement: Supplementary file 1 [file neurosci-07-00080-s001.zip › neurosci-4381676-supplementary.pdf]

Supplementary

Table S1. HbO response of L-C/ R-A condition stimulation compared with baseline measurement.

Channel	Region of Interest	t-value
1	Right superior temporal gyrus (BA 22)	0.12
2	Right angular gyrus (BA 39)	0.82
3	Left middle temporal gyrus (BA 21)	-0.93
4	Right superior temporal gyrus (BA 22)	-0.79
5	Left angular gyrus (BA 39)	0.21
6	Right Middle temporal gyrus (BA 21)	-0.71
7	Left somatosensory association cortex (BA 7)	0.65
8	Right angular gyrus (BA 39)	0.21
9	Right angular gyrus (BA 39)	0.18
10	Right somatosensory association cortex (BA 7)	-0.28
11	Left angular gyrus (BA 39)	1.12
12	Left angular gyrus (BA 39)	0.07
13	Left primary visual cortex (V1, BA 17)	1.11
14	Left somatosensory association cortex (BA 7)	1.62
15	Right somatosensory association cortex (BA 7)	-1.38
16	Left somatosensory association cortex (BA 7)	0.05
17	Right somatosensory association cortex (BA 7)	-0.40
18	Right somatosensory association cortex (BA 7)	-0.23
19	Right superior temporal gyrus (BA 22)	-1.64
20	Left middle temporal gyrus (BA 21)	-1.64
21	Left middle temporal gyrus (BA 21)	-1.53
22	Right superior temporal gyrus (BA 22)	-1.92
23	Right Middle temporal gyrus (BA 21)	-1.32
24	Right Middle temporal gyrus (BA 21)	-2.44
25	Left somatosensory association cortex (BA 7)	-0.23
26	Left somatosensory association cortex (BA 7)	-0.14
27	Right somatosensory association cortex (BA 7)	-1.50
28	Right somatosensory association cortex (BA 7)	0.72
29	Left primary visual cortex (V1, BA 17)	-0.42
30	Left primary visual cortex (V1, BA 17)	-0.13
31	Right primary visual cortex (V1, BA 17)	0.40
32	Right primary visual cortex (V1, BA 17)	-0.14
33	Right Middle temporal gyrus (BA 21)	-1.76
34	Left middle temporal gyrus (BA 21)	-2.18
35	Left primary visual cortex (V1, BA 17)	1.47
